# Access to emerging health technologies in aging: from innovation to inclusion

**DOI:** 10.1016/j.tjfa.2026.100161

**Published:** 2026-05-26

**Authors:** Emanuele Marzetti, Anna Picca, Riccardo Calvani, Hélio José Coelho-Júnior

**Affiliations:** a Fondazione Policlinico Universitario Agostino Gemelli (IRCCS), Largo A. Gemelli 8, Rome 00168, Italy; b Department of Geriatrics, Orthopedics and Rheumatology, Università Cattolica del Sacro Cuore, Largo F. Vito 1, Rome 00168, Italy; c Department of Medicine and Surgery, Libera Università Mediterranea (LUM) Giuseppe Degennaro, Strada Statale 100 km 18, Casamassima 70010, Italy

The rapid expansion of digital and data-driven health technologies has generated considerable optimism for improving the health and independence of older adults. Wearable sensors, telemedicine platforms, artificial intelligence (AI)-enabled risk prediction, and personalized digital interventions hold promise to enhance prevention, enable earlier detection of decline, and support more responsive and individualized care. Yet, as with many innovations in healthcare, their benefits do not appear to be evenly distributed which risks amplifying existing inequities, particularly among vulnerable older adults, who stand to gain the most but are often least well served by current models of access [1,2].

A central challenge is the mismatch between technological design and the lived realities of older individuals. Many digital health tools are developed with younger, technologically competent users in mind. Interfaces may be complex, text small, and interaction pathways unintuitive for individuals with visual impairment, cognitive decline, or reduced dexterity. This issue is particularly relevant in frail, multimorbid older adults in whom functional limitations may impact the ability to engage with technology. For instance, wearable devices that monitor physiological parameters and detect early signs of deterioration are increasingly available, but their uptake among older adults remains limited. Relatively high costs, the need for smartphone integration, and ongoing device management pose tangible barriers [3]. Indeed, programs that have attempted to address these gaps through device provision, simplified interfaces, and community-based training have shown that accessibility is not an inherent feature of technology, but an outcome of design and support [4].

These barriers are readily observable in real-world settings. For instance, among vulnerable older adults receiving community-based services, such as home-delivered meal programs, access to and familiarity with digital tools remain highly variable, with only a proportion reporting internet access or use of connected devices [5]. Similarly, participation in technology-enabled interventions that entail access to devices, connectivity, and baseline digital literacy, may be limited among those at greatest risk of frailty or social isolation [6]. These observations highlight that access is influenced not only by availability, but by broader socioeconomic and functional factors.

Telemedicine offers a similarly illustrative case. Its rapid expansion during and after the COVID-19 pandemic demonstrated clear benefits for older populations, especially for those with mobility limitations or living in underserved areas [7]. However, reliance on video-based platforms has exposed a persistent digital divide. Many older adults lack access to high-speed internet, appropriate devices, or the confidence to engage with virtual care [8]. Evidence from geriatric care models suggests that hybrid and community-supported approaches are more effective in maintaining continuity of care among frail older adults [9]. These examples underscore the broader principle that technological innovation alone is insufficient, unless it is embedded within systems that accommodate diverse capabilities and contexts.

AI-driven tools present both significant opportunity and risk. Predictive algorithms designed to identify frailty, falls risk, or malnutrition could enable earlier and more targeted interventions. Digital biomarkers of frailty are increasingly being explored within geriatric research [10], including approaches that leverage continuous, real-world monitoring of intrinsic capacity and functional trajectories [11]. However, these systems are only as robust as the data on which they are trained. Older adults, especially those who are frail, socially isolated, or from minority and low-resource backgrounds, remain underrepresented in many datasets [12,13]. This introduces the risk of systematic bias, where predictions are less accurate for precisely identifying those individuals most in need of support. Addressing this requires technical solutions, such as more representative data and explainable AI, as well as structural changes to ensure that older populations are meaningfully included in research and development processes [13].

Even when technologies are provided at low cost, sustained engagement depends on usability, perceived relevance, and support. Interventions for vulnerable older adults are more likely to succeed when they incorporate human elements (e.g., regular follow-up, caregiver involvement, integration into existing care relationships), suggesting that technology is most effective when it augments, rather than replaces, interpersonal support. This is reflected in emerging models of remotely delivered interventions, where structured support, whether through clinicians, caregivers, or trained volunteers, improves acceptability and engagement among older adults with frailty [14]. At the same time, evidence suggests that when appropriately designed, technology-based interventions can be both feasible and beneficial in older populations. Digitally delivered exercise and monitoring programs have demonstrated high adherence and meaningful improvements in physical and cognitive outcomes, even among at-risk individuals [15]. These findings emphasize that the key challenge is not whether older adults can engage with technology, but whether technologies are designed inclusively and implemented within supportive environments.

Ethical considerations further complicate implementation. Continuous monitoring technologies can enhance safety and enable early intervention; yet they may also be perceived as intrusive. For older adults, whose priorities often include maintaining independence and quality of life, these tensions are particularly relevant. Frailty-focused care emphasizes person-centered outcomes, including function, autonomy, and well-being. Lessons from long-term care settings further highlight the importance of preserving individuality, dignity, and meaningful engagement in care delivery [16]. Ethical deployment therefore requires a person-centered approach that involves informed consent, transparency, and respect for individual preferences. Co-design methodologies, where older adults are actively involved in shaping technologies, offer a promising pathway to align innovation with user values and expectations [17].

Structural determinants of health must also remain central to this discussion. Frailty is strongly influenced by social determinants, including socioeconomic status, access to care, mobility, and social participation [[18], [19]]. Reduced mobility and social engagement, both common in older populations, can further limit access to both healthcare and enabling technologies. Moreover, periods of enforced isolation, such as during the COVID-19 pandemic, have demonstrated that reduced social interaction and activity can negatively affect health behaviors and accelerate frailty trajectories [20]. While emerging technologies can mitigate some of these effects, they cannot compensate for broader structural inequities. Therefore, ensuring equitable benefit requires alignment between technological innovation and public health strategies, with targeted investment in underserved communities.

From a policy perspective, several practical steps can support more equitable access. Subsidization and reimbursement models can reduce financial barriers, particularly for essential devices and services. Investment in digital infrastructure is foundational. Equally important is the development of age-friendly design standards, ensuring that usability considerations are carefully incorporated into technology development [4]. Training programs for both users and healthcare providers can build confidence and competence, while regulatory frameworks must ensure that commercial offerings meet standards of safety, efficacy, and transparency [12].

Ultimately, the integration of emerging health technologies into geriatric care requires moving beyond a model of innovation driven primarily by capability, towards one guided by inclusivity and real-world applicability. Older adults, especially those living frailty, are a highly heterogeneous group. Recognizing this heterogeneity and designing systems flexible enough to accommodate it are essential for their actual implementation. If employed thoughtfully, emerging health technologies have the potential to support healthier aging, prolong independence, and reduce the burden of frailty. Realizing this potential requires a concerted effort to ensure equitable access, inclusive design, and real-life relevance.

## Funding

None.

## Declaration of generative AI and AI-assisted technologies in the writing process

During the preparation of this manuscript, the authors used ChatGPT-5.2 to assist with language refinement only. The authors reviewed and edited all content and take full responsibility for the accuracy, integrity, and originality of the work.

## CRediT authorship contribution statement

**Emanuele Marzetti:** Conceptualization, Writing – original draft. **Anna Picca:** Conceptualization, Writing – review & editing. **Riccardo Calvani:** Conceptualization, Writing – review & editing. **Hélio José Coelho-Júnior:** Conceptualization, Writing – review & editing.

## Declaration of competing interest

The authors declare that they have no known competing financial interests or personal relationships that could have appeared to influence the work reported in this paper.
